# Genetic approaches in improving biotechnological production of taxanes: An update

**DOI:** 10.3389/fpls.2023.1100228

**Published:** 2023-01-26

**Authors:** Edgar Perez-Matas, Diego Hidalgo-Martinez, Ainoa Escrich, Miguel Angel Alcalde, Elisabeth Moyano, Mercedes Bonfill, Javier Palazon

**Affiliations:** ^1^ Department of Biology, Healthcare and the Environment, Faculty of Pharmacy and Food Sciences, University of Barcelona, Barcelona, Spain; ^2^ Departament de Medicina i Ciències de la Vida (MELIS), Universitat Pompeu Fabra, Barcelona, Spain

**Keywords:** paclitaxel, *Taxus* spp., genetic transformation, metabolic engineering, synthetic biology

## Abstract

Paclitaxel (PTX) and its derivatives are diterpene alkaloids widely used as chemotherapeutic agents in the treatment of various types of cancer. Due to the scarcity of PTX in nature, its production in cell cultures and plant organs is a major challenge for plant biotechnology. Although significant advances have been made in this field through the development of metabolic engineering and synthetic biology techniques, production levels remain insufficient to meet the current market demand for these powerful anticancer drugs. A key stumbling block is the difficulty of genetically transforming the gymnosperm *Taxus* spp. This review focuses on the progress made in improving taxane production through genetic engineering techniques. These include the overexpression of limiting genes in the taxane biosynthetic pathway and transcription factors involved in its regulation in *Taxus* spp. cell cultures and transformed roots, as well as the development and optimization of transformation techniques. Attempts to produce taxanes in heterologous organisms such as bacteria and yeasts are also described. Although promising results have been reported, the transfer of the entire PTX metabolic route has not been possible to date, and taxane biosynthesis is still restricted to *Taxus* cells and some endophytic fungi. The development of a synthetic organism other than *Taxus* cells capable of biotechnologically producing PTX will probably have to wait until the complete elucidation of its metabolic pathway.

## 1 Introduction

Plant cell, tissue, and organ *in vitro* cultures are excellent platforms to produce specialized compounds of interest for the chemical/pharmaceutical industries, such as ginsenosides (Mallol et al., 2001), tropane alkaloids like scopolamine (Palazon et al., 2003), ginkgolides (Sabater-Jara et al., 2013), *trans*-resveratrol (Almagro et al., 2022) or centellosides (Alcalde et al., 2022a), among others. Another important compound produced by means of these biotechnological tools is paclitaxel (PXT), which was approved by the Food and Drug Administration (FDA) for the treatment of refractory metastatic ovarian cancer in 1992, and metastatic breast cancer (refractory or insensitive to anthracyclines) in 1994. The highly positive results obtained with PTX have extended its use to non-small cell lung, prostate, stomach, cervical, esophageal, testicular, and pancreatic cancers, as well as AIDS-related Kaposi’s sarcoma and leukopenia (Perez-Matas et al., 2022). Additionally, PTX is now under study for the treatment of neurodegenerative disorders such as Alzheimer’s or Parkinson’s disease (Zhang et al., 2005) and other health conditions related with the stabilization of microtubule-associated proteins such as psoriasis (Ehrlich et al., 2004).

Due to its effectivity, PTX is perhaps the most important antitumor agent in history. In 2021, the global PTX market was valued at US$ 4.51 billion and is expected to reach over US$ 11.16 billion by 2030 (Perez-Matas et al., 2022). More than 20 companies in several countries are involved in the development and commercialization of new pharmaceutical forms of PTX, most notably in the US, which accounts for almost 40% of the global oncology market (Sofias et al., 2017). Obtaining this compound or a semisynthetic precursor from *Taxus* trees is ecologically unsustainable due to their low natural levels, and chemical synthesis is economically unfeasible. Thus, the biotechnological production of PTX through optimized *Taxus* spp. cell cultures is the current method of choice because cell suspensions can be grown under controlled and cost-effective conditions. For instance, Plant Cell Fermentation (PCF^®^) technology has been implemented by Phyton Biotech, the world’s largest nowadays supplier of PTX (Perez-Matas et al., 2022). Despite this, the PTX yields of biotechnological production platforms remain low. As a result, PTX is one of the most expensive and highest added-value compounds on the market. Although the PTX biosynthetic pathway is not completely understood, the main flux-limiting steps have been elucidated, opening opportunities for a metabolic engineering approach to increasing its production or that of its semisynthetic precursors. One of the main hurdles in the application of metabolic engineering techniques in *Taxus* spp. cell/organ cultures is the difficulty of genetically transforming these gymnosperm plants, and the slow growth of the transformed plant material under *in vitro* conditions. For these reasons, the genetic transformation of *Taxus* spp. remains an important challenge for plant biotechnology.

This review examines the progress made in the genetic transformation of *Taxus*, focusing on key studies that have used a wide range of *Taxus* species, plant material, transformation systems, vectors, and culture conditions. Successful applications of metabolic engineering in this field are summarized. Other aspects covered are the heterologous expression of genes involved in the PTX biosynthetic pathway and the potential of synthetic biology to develop a rapid and straightforward PTX production system.

## 2 Progress in the genetic transformation of *Taxus* spp.

**Figure 1:**
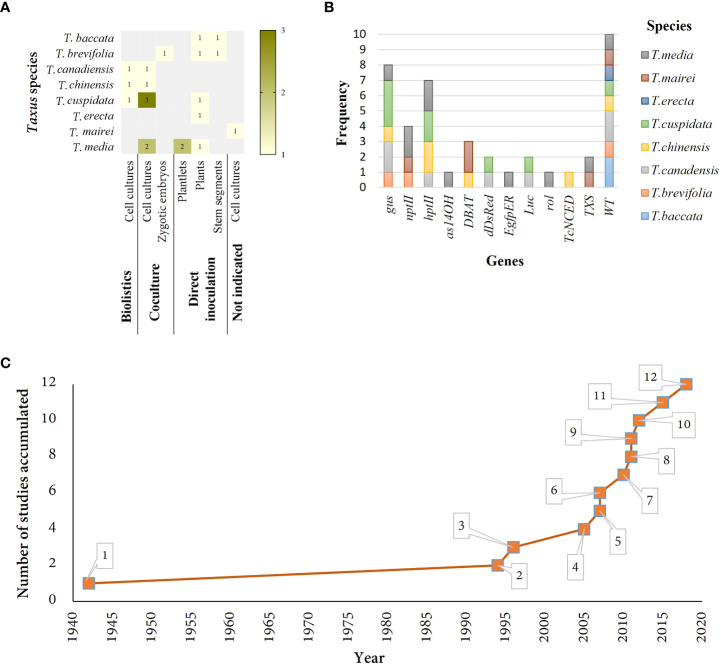
Summary of studies related to *Taxus* cell cultures. **(A)** Studies in different Taxus species grouped by genetic transformation technique and plant material used. **(B)** Frequency of exogenous genes used across different species of taxus. **(C)** Number of studies accumulated in *Taxus* cell cultures through time. 1. Direct inoculation of plants resulted in crown galls (Smith, 1942). 2. Direct inoculation of stem segments resulted in crown galls (Han et al., 1994). 3. GUS activity was observed after coculture of plasmid and Zygotic embryos (Luan et al., 1996). 4. Stable cell line overexpressing *S-DBAT* gene (Ho et al., 2005). 5. Stable transformants after infection with Agrobacterium (Ketchum et al., 2007). 6. Transient transformation achieved with biolistic on cell cultures. Elicitation experiments tested. (Vongpaseuth et al., 2007). 7. Higher production of PTX in cell lines with *rol* genes, overexpressing *TXS* gene (Exposito et al., 2010). 8. Stable cell line overexpressing *DBAT* gene (Zhang et al., 2011b). 9. Decreased of C14OH taxanes on transgenic cell lines overexpressing *as14OH* gene (Li et al., 2011). 10. Cell lines overexpressing *TcNCED* gene and transformed by biolistic showed higher levels of ABA and PTX (Li et al., 2012). 11. The highest transformation efficiency (75%) achieved in *T. x media* cell cultures (Martínez-Márquez et al., 2015). 12. Identification of best *Agrobacterium* strain for cell transformation of *T.cuspidata* and *T. canadensis* (Wilson et al., 2018).

**Figure 2:**
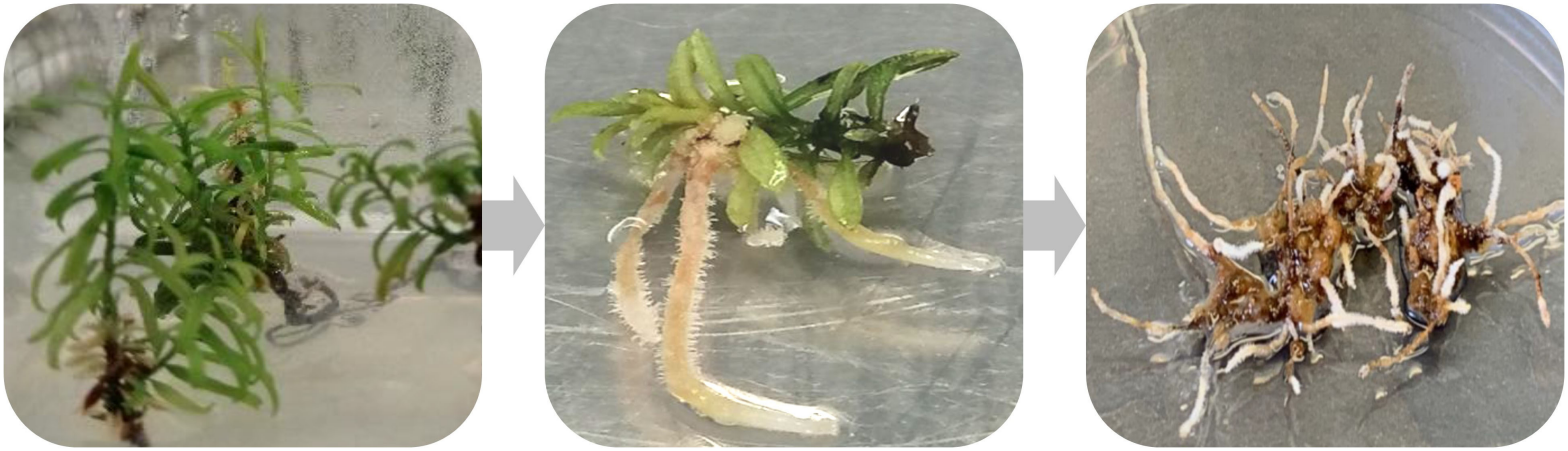
Summarized process of Taxus hairy roots induction and culture. The left image shows 8-week-old T. baccata seedlings grown in hormone-free DCR medium, these seedling were infected with Agrobacterium rhizogenes. The middle image shows the development of hairy roots at the infection site. The right image shows a stable, 1-year-old hairy root line isolated from the infected explant.

There have been many attempts to obtain transformed cell (**Table 1**, **Figure 1**) or hairy root cultures (**Table 2**, **Figure 2**) of *Taxus* species using very different methodologies and strategies (**Figures 1A, B**), but few research groups have been successful (**Figure 1C**). As mentioned, the slow and difficult *in vitro* culture of these gymnosperm plants is a serious obstacle for developing highly productive transgenic production platforms of PTX or its semi-synthetic precursors.

**Table 1:** Transformed *Taxus* cell cultures.

Plants	Bacteria	Genes included	Transf. system	Plant material	Results	PTX Production	Reference
*T.baccata*, *T.erecta, T.brevifolia, T.cuspidata* *T.media*	*Phytomonas tumefaciens*	Wild type	Direct Inoculation	Plants	Crown galls	Not studied	(Smith, 1942)
*T.brevifolia* *T.baccata*	*A. tumefaciens* (Bo542, C58)	Wild type	Direct Inoculation	Stem segments	Crown galls	Same PTX as control	(Han et al., 1994; Han et al., 2000)
*T.brevifolia*	**-**	*gus, nptII*	Coculture: plasmid-embryos	Zygotic embryos	GUS activity in mature seed embryos	Not studied	(Luan et al., 1996)
*T.cuspidata*	*A.rhizogenes* ATCC 15834 *A.tumefaciens* EHA105	*gus, hptII*	Coculture: Bacteria-plant cells	Cell cultures	Stable trans. 1% T.E. Good growth	Same PTX and taxane pattern as Control	(Ketchum et al., 2007)
*T.cuspidata* *T.canadensis*	–	*gus, Luc, dDsRed*	Biolistic	Cell cultures	Transient transformation	Success of different promoters	(Vongpaseuth et al., 2007)
*T.mairei*	Not indicated	*S-DBAT, AS-DBAT, AS-TXS, nptII*	Not indicated	Leaves, Cell cultures	Stable cell line with *S-DBAT*	Need of MeJa. for x 2.5 more PTX and baccIII.	(Ho et al., 2005)
*T.chinensis*	*A. tumefaciens* LBA4404	*hptII, gus/* *hptII, gus, DBAT*	Coculture: Bacteria-plant cells	Cell cultures	Cell line overexpressing *DBAT* gene	x 1.7 more PTX, without MeJa	(Zhang et al., 2011b)
*T. x media*	*A. tumefaciens* GV3101	*hptII, nptII, gus/hptII, nptII, gus, as14OH*	Coculture: Bacteria-plant cells	Cell cultures	Transgenic cell lines with or without the *as14OH* gene	Decrease of C14OH taxanes	(Li et al., 2011)
*T.chinensis*	–	*TcNCED* *hptII*	Biolistics	Cell cultures	Cell lines overexpressing Tc*NCED* gene	Increase 48% ABA; 2.7- times more PTX	(Li et al., 2012)
*T. x media*	*A. tumefaciens* C58C1 *A. rhizogenes* LBA 9402	*rol* genes + *TXS, hptII* *rol* genes	Direct inoculation	Plantlets	Cell lines from dedifferentiated root lines with *rol* genes, overexpressing or not *TXS* gene	Elicited TXS cell line 256% and 176% higher than the elicited untransformed and rolC cell lines, respectively.	(Exposito et al., 2010)
*T. x media*	*A.tumefaciens* C58C1	*nptII, EgfpER*	Coculture: bacteria – plant cells	Cell cultures	75% T.E.	Not studied	(Martínez-Márquez et al., 2015)
*T.cuspidata* *T.canadensis*	*A. tumefaciens* C58C1, EHA105, GV3101, LBA4404	*hptII, gus*	Coculture: bacteria-plant cells	Cell cultures	Best strain: EHA105. Transf. lines >5 years. Improved method	Not studied	(Wilson et al., 2018)

PTX, paclitaxel; gus, β-glucuronidase; T.E., transformation efficiency; nptII, neomycin phosphotransferase II; hptII, hygromycin phosphotransferase II; luc, luciferase; dDsRed, red fluorescent protein; MeJa, methyl jasmonate; bacIII, baccatin III; AS/S-DBAT, antisense/sense-10-deacetyl-baccatin III-10-O-acetyltransferase; AS-TXS, antisense-taxadiene synthase; as14OH, antisense-taxane 14β-hydroxylase; TcNCED, Taxus chinensis 9-cis-epoxycarotenoid dioxygenase; ABA, abscisic acid; EgfpER, green fluorescent protein.

**Table 2:** *Taxus* hairy root cultures.

Plants	Bacteria	Constructed Plasmid/DNA	Genes included	Transf. System	Plant material	Results	PTX Production	Reference
*T. x media*	*A. rhizogenes* ATCC 15834, TR 105 and LBA 9402	Wild type	*rol* genes	Direct inoculation	Plantlets	Best strain: LBA9402. 3% transformation frequency	210 μg/g D.W. in MeJa elicited cultures 568,2 μg/L In MeJa + Phen treated cultures 3179,9 mg/gDW in primed with BABA and elicited with MIX cultures	(Furmanowa and Syklowska-Baranek, 2000) (Syklowska-Baranek et al., 2009) (Sykłowska-Baranek et al., 2022)
*T. cuspidata*	*A. rhizogenes* ATCC15834, R1000 and A4	Wild type	*rol* genes	Direct infection, liquid co-culture, solid coculture	3-weeks old seedlings	Three stable root lines were obtained. Culture conditions were optimized	52.6 mg/L in the best root line in optimum conditions elicited with MeJa	(Kim et al., 2009)
*T. x media*	*A rhizogenes* C58C1	pRiA4 + pCAMBIA -TXS-His	*rol* genes, *TXS* and *hptII*	Direct inoculation	Plantlets	Hairy root line ATMA overexpressing TXS gene	10.78 mg/L in MeJa + Phen treated cultures 1440.8 μg/g DW in MeJa elicited + PDF degassed cultures 1434.9 μg/g DW in MeJa + Viscozym + PFD degassed treated cultures 2473.29 μg/g DW in MeJa + PFD degassed treated cultures	(Sykłowska-Baranek et al., 2015a) (Sykłowska-Baranek et al., 2019b) (Sykłowska-Baranek et al., 2018) (Sykłowska-Baranek et al., 2019a)
*T. baccata*	*A. rhizogenes*	Wild type	*rol* genes	Co-culture	Acclimated plants	34% transformation efficiency with ultrasounds and heating explants	Higher taxol than the control (spectroscopically measured)	(Sahai and Sinha, 2022)

MeJa, methyl jasmonate; Phen, phenylalanine; BABA, β-aminobutyric acid; MIX, MeJa + sodium nitroprusside + Phen elicitation treatment; TXS, taxadiene synthase; PDF, perfluorodecalin; hptII, hygromycin phosphotransferase II; PTX, paclitaxel.

### 2.1 Transformed *Taxus* cell cultures

One of the earliest efforts to transform *Taxus* spp. was by Smith (1942), who used *Phytomonas tumefaciens* to artificially inoculate plants of *T. baccata* Erecta Loud, *T. brevifolia* Nutt, *T. cuspidata* Sieb. and Zucc. and *T. media* Rehd., grown in controlled conditions. Most of the plants developed crown galls with variable growth capacity. (Han et al, 1994; Han et al, 2000) genetically transformed stem segments of mature *T. brevifolia* and *T. baccata* trees with *Agrobacterium tumefaciens* (Bo542 and C58). Although the obtained gall cell lines grew well, the PTX production (μg/g dry weight) of untransformed calli was not improved.

Transient transformation was assayed by Luan et al. (1996), who incubated zygotic embryos with the pB112 expression vector carrying the β-glucuronidase (GUS) gene and the kanamycin resistance gene (*nptII* gene) under the control of the cauliflower mosaic virus 35S (CaMV35S). GUS expression was achieved in 92% of the mature embryos, indicating that the efficiency of the transformation depended on the developmental stage of the embryos.

The group of Croteau, one of the most outstanding in the field of PTX production in *Taxus* cell cultures, have also dedicated efforts to obtain genetically transformed cell cultures using the *Agrobacterium* system (Ketchum et al., 2007). They successfully established transformed hygromycin-resistant *T. cuspidata* cell cultures carrying the GUS reporter gene, achieving sufficient biomass after 20 months to establish a bioreactor culture. The genetic transformation did not alter the levels and pattern of taxane production. With the aim of characterizing the genes involved in PTX biosynthesis and its regulation, Vongpaseuth and Roberts, 2007 achieved a particle bombardment-mediated transient transformation of *T. cuspidata* and *T. canadensis* with a firefly luciferase gene under the control of the constitutive CaMV35S promoter. Parameter optimization, however, appears to be highly dependent on the cell line used.

Metabolic engineering is a very useful technology as it allows the manipulation of endogenous metabolic pathways of a wide variety of secondary compounds. The transfer and integration of genes encoding enzymes involved in limiting metabolic steps generally boosts the endogenous production of compounds through the target pathway. The application of this methodology has allowed the enhancement of PTX production in *in vitro* cultures capable of overexpressing key or limiting biosynthetic genes.

With the aim of improving the biotechnological production of taxanes, transgenic cell cultures of *Taxus mairei* constitutively harboring the gene encoding the enzyme 10-deacetyl baccatin III-10-O-actyl transferase (DBAT) were obtained. However, taxane production remained dependent on elicitation with methyl jasmonate (MeJa) in the transgenic root lines, only one of which achieved a high PTX yields (Ho et al., 2005). In 2011, Zhang et al. (2011a) obtained transgenic *Taxus chinensis* cells also overexpressing the *DBAT* gene after coculture with an *A. tumefaciens* strain. In this case, the PTX production of the transformed cells was 1.7-fold higher compared to the untransformed cells without the addition of MeJa.

Another metabolic strategy used in *T. x media* cell cultures has been to block the branch points in PTX biosynthesis by antisense-induced suppression of the taxane-14-hydroxylase gene, whose product catalyzes the biosynthesis of oxygenated taxanes at C14. As these compounds compete with PTX for the same initial precursors, blocking their formation led to increased PTX production in transgenic cell lines (Li et al., 2011).

On the other hand, ozone is known to induce taxane production in *T. chinensis* cell cultures and the response is at least partially dependent on abscisic acid (ABA) signaling (Xu et al., 2011). Applying this strategy, transgenic *T. chinensis* cell lines were obtained through bombardment with particles carrying a plasmid vector harboring the *TcNCED1* and *hpt* genes under the control of the 35S promoter. The *NCED* gene encodes 9-cis-epoxycarotenoid dioxygenase, the enzyme responsible for the cleavage of 9-cis-epoxycarotenoid, a rate-limiting step in ABA biosynthesis. ABA accumulation in the transgenic cell lines increased by 48% and PTX production was 2.7-fold higher compared to the untransformed cells (Li et al., 2012).

Exposito et al. (2010) obtained transformed root cultures of *T. media* overexpressing the *T. baccata TXS* gene, which encodes the enzyme controlling the first step specific to taxane biosynthesis. As the root lines had a low growth rate, two were selected, both containing the *A. rhizogenes rol* genes and only one with the *TXS* gene. Plant growth regulators were then applied to dedifferentiate the roots and the calli obtained were used to establish suspension cell cultures. Three *T. x media* cell lines were developed, an untransformed control, the rolC line (carrying the *rol* genes), and the TXS line (carrying both *rol* and *TXS* genes), and used to establish a two-stage culture system. In the first stage, cells were cultured in growth medium optimized for biomass production for 12 days. Thereafter they were transferred to a production medium optimized for taxane production, with or without the elicitor MeJa, and maintained for 28 days. The taxane production of the TXS line was found to be 2.64-fold higher compared to the control and 1.55-fold higher than the rolC line, all in elicited conditions.

Martínez-Márquez et al. (2015) described a reliable protocol for the stable transformation of *T. x media* cell cultures. Ten transformed calli were obtained from 1 g fresh weight of plated *Taxus* cells, 75% of which were maintained for several months in a continuous selection medium. Other efficient method for the genetic transformation of *T. cuspidata* and *T. canadensis* calli and cell suspensions with an optimal *A. tumefaciens* strain (EHA105) was described by Wilson et al. (2018). The transformed calli showed a stable GUS expression for more than five years. This technology represents a clear improvement over other methods for *Taxus cell* transformation with *A. tumefaciens*.

### 2.2 *Taxus* hairy root cultures

Hairy root cultures are a very useful biotechnological platform for the production of natural products, mainly those that are synthesized in the roots of the original plant. These cultures are established after the infection of plants with different strains of *A. rhizogenes*. Hairy root cultures present several advantages over other biotechnological systems, including their relatively fast growth rates (in hormone-free media), genetic and biochemical stability, and capacity for organogenesis-associated synthesis of metabolites (Alcalde et al., 2022b). In addition, hairy root cultures may be scaled up from small-scale systems to large-scale industrial processes.

However, obtaining hairy root cultures of *Taxus* spp. can be very difficult due to low transformation efficiency, the length of time between infection and hairy root formation, and the low growth rate of *Taxus* hairy roots (**Figure 2**). Nevertheless, several research groups have achieved hairy root cultures with improved growth and production as mention below.

In 2000, Furmanowa and Syklowska-Baranek (2000) and Furmanowa et al. (2000) successfully established transformed root cultures of *T. x media* by directly infecting several kinds of explants with three different strains of *A. rhizogenes*. The hairy roots appeared 19 weeks after inoculation and grew slowly for one and a half years, after which a stable culture was achieved. The PTX production of these roots after three weeks of elicitation with MeJa was three-fold higher compared to the control roots. The same research group reported that the addition of the precursors L-phenylalanine and p-aminobenzoic acid to the culture medium significantly increased taxane production. Notably, the addition of L-phenylalanine together with MeJa increased PTX production 14-fold compared to untreated cultures (Syklowska-Baranek et al., 2009).

Sykłowska-Baranek et al. (2022) recently described an efficient strategy that clearly increased PTX production of *T. x media* hairy roots. After pretreatment with 100 μM β-aminobutyric acid for one week, the cultures were elicited with 100 μM MeJa, 10 μM nitroprusside and 100 μM L-phenylalanine. After 14 days of elicitation, the PTX production was 3179.9 μg/g dry weight, which was 613-fold higher than in control cultures.

*T. cuspidata* hairy root cultures were obtained by Kim et al. (2009) after infecting three-week old *Taxus* seedlings with three wild strains of *A. rhizogenes* (ATCC 15834, R1000, and A4), following three different methodologies: direct infection, liquid co-culture and solid co-culture. Three stable root lines were obtained after 10 months of subculture and the best line was selected for optimization of the culture conditions, resulting in a PTX yield of 52.6 mg/L after 14 days of elicitation with MeJa.

The *T. media* hairy root cultures established by Exposito et al. (2010) were obtained by directly infecting plantlets with the *A. rhizogenes* LBA 9402 wild strain or *A. tumefaciens* C58C1 carrying the *A. rhizogenes* RiA4 plasmid and binary plasmid pCA-TXS-His harboring the *TXS* gene of *T. baccata*. The transformation was performed under the control of the CaMV35S promoter and using the hygromycin phosphotransferase gene (*hptII*) as a resistance marker. The transformed nature of the root lines was confirmed by PCR analysis, but due to poor root growth, as mentioned above, the hairy root lines were dedifferentiated, and *T. media* cell cultures were established with the *rol* genes and with or without the *TXS* gene.

In 2015, Sykłowska-Baranek et al. (2015b) obtained *T. x media* transgenic roots overexpressing the *TXS* gene, whose growth and taxane production were considerably improved in two-phase liquid cultures with aerated or degassed perfluorodecalin (PFD). The addition of MeJa (100 μM) to the cultures increased PTX production, whereas supplementation with coronatine (1 μM) was more beneficial for baccatin III accumulation. The expression of several PTX biosynthetic genes was always higher in the *TXS*-overexpressing versus wild type hairy roots, as was the taxane production (Sykłowska-Baranek et al., 2019a). The same research team also studied PTX production and phenylalanine ammonia-lyase (PAL) activity in two *T. x media* hairy root lines overexpressing the *TXS* gene (Sykłowska-Baranek et al., 2015a). After elicitation with MeJa (100 μM) and phenylalanine (100 μM), the highest PTX yield was associated with maximum PAL activity in one hairy root line, but not in another.

A biotechnological system based on the addition of cellulase to the *T. x media* hairy root cultures overexpressing the *TXS* gene clearly enhanced PTX release from the producer cells to the medium (Sykłowska-Baranek et al., 2018), although the total production did not increase. (For a review, see Sykłowska-Baranek et al. (2019b).

In 2022, Sahai and Sinha (2022) established *T. baccata* subsp. *wallichiana* hairy roots after co-culturing explants of acclimated trees with *A. rhizogenes*, with the addition of 100 μM acetosyringone and applying sonication (30 kHz, 300 W for 60 s at 5 s intervals) and heating (4°C for 5 min). Transformed roots emerged after seven days of co-cultivation, and 34% of the explants with hairy roots were transformed. After 4 months of growth, the PTX production of the hairy roots, spectroscopically measured, was higher than the control.

The results of all these studies confirm the possibility of establishing transformed cell or hairy root cultures and the efficacy of metabolic engineering approaches in increasing the production of PTX and related taxanes in *Taxus* spp. Further studies are needed to continue improving the yields of PTX and its derivatives to meet the growing clinical/industrial demand for these drugs.

### 2.3 Other transformation systems

Endophytic fungi are able to produce PTX, mainly if they are living with their host *Taxus* species. A study combining phytochemistry, molecular biology and genome sequencing failed to detect the PTX pathway or biosynthetic genes in fungal endophytes associated with *Taxus* spp. (Heinig et al., 2013). Nevertheless, since the first reports by Stierle et al. (1993) and Strobel et al. (1996), several studies have confirmed taxane production by endophytic fungi of *Taxus* and other plant species (for reviews, see Zhou et al. (2010); Shankar Naik (2019)). In fact, approximately 200 different fungi representing diverse orders are thought to produce PTX (Flores-Bustamante et al., 2010; Swamy et al., 2022), albeit in very low and unstable levels.

The low contents of PTX and related taxanes in endophytic fungal cultures, and the decrease in production over successive subcultures have inspired several attempts to increase PTX yields by genetically transforming the fungi. In 2007, Wang et al. (2007) transformed the fungus BT2 isolated from *T. chinensis* var. *mairei* using restriction enzyme-mediated integration technology. The plasmid pV2 used for the fungal protoplast transformation harbored hygromycin B and the ampicillin resistance gene as selective markers. Another transformation system of fungal protoplasts was described by Wei et al. (2010). In this case the PTX-producing endophytic fungus was *Ozonium* sp. EFY-21 and the genetic transformation was mediated by polyethylene glycol (PEG) with the same pV2 plasmid, but carrying the hygromycin-B phosphotransferase gene under the control of the fungal promoter trpC. The frequency of protoplast regeneration was higher than 6%. The success of these two different transformation methodologies opened the possibility of transferring PTX biosynthetic genes to improve fungal taxane production.

Zhang et al. (2011b) stably transformed spores of the PTX-producing fungus *Cladosporium cladosporioides* MD2 with *A. tumefaciens* LBA4404 carrying the binary vector pCAMBIA1303, which harbored the hygromycin-resistance gene under the control of the CaMV35S promoter and the Nos terminal. Optimal co-culture conditions were established for an efficient and stable fungus transformation. Two years later, Liu et al. (2013) developed an efficient protocol for transforming the PTX-producing endophytic fungus *Ozonium* sp. EFY21 with *A. tumefaciens* EHA 105 carrying the expression vector pCAMBIA 1304’AN7-1 and the hygromycin-resistance gene. Several factors that affect transformation and transformant stability were also optimized. However, neither of these studies integrated PTX biosynthetic genes into the genome of the transformed fungi.

The CRISPR/Cas9 system has emerged as a powerful and precise tool for genome engineering in various organisms, including filamentous fungi, in which the genome has been edited with remarkable success. PTX production in filamentous fungi could potentially be enhanced by applying this technology to block the sterol metabolic pathway by knocking out squalene synthase and lanosterol synthase and channeling the precursors toward PTX synthesis (El-Sayed et al., 2017). Although it is still premature to proclaim that this genome editing system has ushered in a golden age for the production of pharmaceutical products in manipulated fungi, it is certainly a promising avenue for the future.

Another transformation strategy aimed at understanding the molecular mechanisms involved in taxane production was described by Sanchez-Muñoz et al. (2020). Transient PEG-assisted transformation of *T. x media* protoplasts was achieved with the transcription factors BIS2 from *Catharanthus roseus* (Van Moerkercke et al., 2016) and TSAR2 from *Medicago truncatula* (Mertens et al., 2016) cloned into the PK7WG2D plasmid. The expression of several taxane-related genes was subsequently found to be upregulated, indicating that this approach, combined or not with elicitation, could serve to establish more efficient *Taxus* spp. transformed systems with increased PTX production rates.

Recently, Kashani et al. (2022) transiently transformed *T. baccata* leaves by overexpressing the full length of the DBTNBT coding sequence using an *A. tumefaciens* LBA 4404 vacuum infiltration method. The DBTNBT enzyme controls the last step of the PTX biosynthetic pathway. Two *A. tumefaciens* strains were used, carrying the vectors pCAMBIA-DBTNBT or (without the *DBTNBT* gene) pCAMBIA1304. The overexpression of this gene clearly increased the PTX production in relation to the controls, being 7.4-fold higher when coronatine (1 μM) and cyclodextrins (50 mg/L) were added to the incubation cultures.

## 3 Agrobacteria and expression vectors used for genetic transformation

The genetic transformation of plant species, widely used in the field of plant biotechnology, allows foreign genes to be inserted into the recipient plant tissue to create genetically modified cultures. The gene transfer methods most frequently used in plants are the *Agrobacterium* spp. system, PEG, electroporation, and biolistic technology (Rakoczy-Trojanowska, 2002). The main impediment to genetically modifying *Taxus* spp. is the lack of an efficient and reproducible transformation system. The advantages of using the *Agrobacterium* spp. to infect diverse groups of plant species are well documented, but transgene expression remains difficult in gymnosperms, even when supervirulent strains are used (Van Der Fits et al., 2000; Gelvin, 2003). Transformation of recalcitrant conifers by particle bombardment (biolistic) has been used to introduce both linear and plasmid DNA constructs, but few publications report transient or stable transformation of *Taxus* spp. Other obstacles are the difficulty of gymnosperm plant regeneration after genetic transformation, and the probable appearance of fragmented or multiple copy transformation events that lead to transgene silencing.

*Agrobacterium* is a genus of bacteria that induce the growth of tumors or rhizogenesis in plants by a natural ability to transfer DNA to plant cells. This mechanism has been exploited as a biotechnological tool and the *Agrobacterium* system is the most used for the genetic transformation of plants. After the recent reclassification of *Agrobacterium* species (Flores-Félix et al., 2020), most are now included in the genus *Rhizobium* and others in *Ruegeria*, *Pseudorhodobacter* and *Stappia* (new genera). Thus, the most used agrobacteria in plant biotechnology are currently known as *Rhizobium radiobacter*, *R. rhizogenes*, *R. rubi* and *R. vitis*, respectively. The latter two species induce tumors/crown galls in several plants.

## 4 Taxane production in heterologous systems: Synthetic biology

Despite certain outstanding successes in the genetic transformation of *Taxus* spp., as outlined above, this process has been scarcely reported compared with the number of studies on the genetic transformation of angiosperm plants, both monocots and dicots. Moreover, the PTX production achieved in genetically transformed cultures remains relatively low. This has prompted investigation into the potential of metabolic engineering of non-taxane-producing heterologous systems. The development of synthetic biology techniques has created a hopeful scenario for establishing alternative high-yielding production systems for PTX and related taxanes.

Model organisms used for the insertion and expression of target biosynthetic genes offer the advantages of being easier to work with and scale up. They include microorganisms such as *Escherichia coli*, and *Saccharomyces cerevisiae* (Vongpaseuth and Roberts, 2007). Plants such as *Arabidopsis thaliana* and *Nicotiana* sp. are used for ectopic genetic transformation as they are readily transformed and possess all the metabolic processes typical of higher plants.

### 4.1 Metabolic engineering in *E. coli* and other bacteria

One of the first attempts to obtain taxanes in a non-PTX-producing organism used a prokaryotic system based on *E. coli* (Huang et al., 2001). Four genes related to the taxane biosynthetic pathway were co-expressed: two specific genes encoding taxadiene synthase (TXS) of *T. brevifolia* and geranylgeranyl diphosphate synthase (GGPPS) from *Erwinia herbicola* and two general terpene biosynthetic genes encoding isopentenyl diphosphate synthase from *Schizosaccharomyces pombe* and 1 deoxy-D-xylulose 5-phosphate synthase (DXS) endogenously expressed in *E. coli.* The successful expression of these four transgenes in *E. coli* resulted in the production of 1.3 mg/L of taxadiene (Huang et al., 2001), a far lower amount than obtained in *Taxus* spp. cell cultures. Although the ultimate aim would be to reconstruct the entire PTX biosynthetic pathway in *E. coli*, introducing complex eukaryotic metabolism in a prokaryotic model is proving extremely difficult.

Using a multivariate-modular strategy, taxadiene production in *E. coli* was improved about 15000-fold (1 g/L) by engineering the native methylerythritol-phosphate (MEP) pathway (Ajikumar et al., 2010). The first module comprised an upstream MEP pathway with eight genes, and the expression of four genes thought to be rate-limiting was modulated. The second module consisted of a two-gene downstream heterologous pathway to taxadiene. The P450-based oxidation system of PTX biosynthesis in *E. coli* was then engineered to convert taxadiene to taxadien-5α-ol, providing the basis for the synthesis of subsequent metabolites by means of similar cytochrome P450 (CYP450) oxidation. The optimized engineered strain improved taxadiene-5α-ol production 2400-fold compared to the state of the art with yeast. The study revealed that taxadiene synthesis is severely inhibited by exogenous indole at levels higher than ~100 mg/L. Further increases in the indole concentration also inhibited cell growth, with the degree of inhibition being highly strain-dependent. Although the biochemical mechanism of indole interaction with the isoprenoid pathway is presently unclear, the results suggest a possible synergistic effect between indole and terpenoid compounds of the isoprenoid pathway that inhibits cell growth. In general, functional expression of plant CYP725A4 in *E. coli* (Edgar et al., 2017) is challenging because of the inherent limitations of bacterial platforms, such as the absence of electron transfer machinery and CYP450-reductases (CPRs), and the translational incompatibility of the membrane signal modules of CYP450 enzymes due to the lack of an endoplasmic reticulum.

In the same year as the previous study, Bian et al. (2017) established the mevalonate pathway and the *TXS* gene in *E.coli*. The engineered bacteria were able to produce taxadiene, although in low quantities. Additionally, they achieved the first transfer of a taxadiene-producing platform from bacteria to the filamentous fungus *Alternaria alternata*. Several genes related to post-translational modifications, including those expressing cytochrome P450, were easily introduced into the fungus using the *Agrobacterium* system. Transformation efficiency and taxadiene levels were increased when strong heterologous promoters were used.

In a less challenging approach, *E. coli* has been harnessed to produce taxane intermediates for semi-synthesis. Loncaric et al. (2006) obtained baccatin III in transgenic *E. coli* that expresses the *DBAT* gene and is capable of acylating the added exogenous substrate, 10-deacetylbaccatin III.

Another taxadiene-producing microorganism host, *Bacillus subtilis* 168, was obtained for the first time by Abdallah et al. (2019). It was metabolically engineered to overexpress the plant-derived *TXS* gene and a synthetic operon harboring the *B. subtilis* genes involved in the MEP pathway together with the *IspA* gene (encoding the geranyl and farnesyl pyrophosphate synthases). Moreover, a vector carrying the *crtE* gene (encoding GGPPS) was introduced to increase the supply of GGPP. The overexpression of the MEP pathway enzymes along with *IspA* and *GGPPS* genes resulted in an 83-fold increase in taxadiene production compared to the strain expressing only *TXS* and relying on the innate pathway of *B. subtilis*. The total amount of taxadiene produced by the latter engineered strain was 17.8 mg/L, indicating that *B. subtilis* could also be a good platform for PTX production.

Nevertheless, the use of *E. coli* and other prokaryotic cells as heterologous production systems of plant metabolites has several limitations arising from the absence of an efficient isoprenoid biosynthetic pathway. Moreover, prokaryote hosts have a tendency to produce the target proteins in an insoluble and non-functional form, and have a limited supply of NADPH:cytochrome P450 reductase, which is essential for the correct function of plant cytochromes P450 (Julsing et al., 2006). In addition, prokaryotes lack the compartmentalization of eukaryotic cells, which creates different intracellular environments that allow spatial or temporal portioning of intermediary metabolites during the formation of the final product. For these reasons, eukaryotic unicellular organisms such as yeasts are attracting attention, as they feature the typical membrane-enclosed organelles of eukaryotes and cell compartmentalization, an essential requirement for the biosynthesis of most specialized compounds.

### 4.2 Metabolic engineering in yeast: *Saccharomyces cerevisiae*

DeJong et al. (2006) produced the PTX intermediate taxa-4(20),11(12)-dien-5α-10β-ol in *S. cerevisiae* transformed with eight of the 19 known PTX biosynthetic genes (*GGPPS*, *TXS*, *T5αOH*, *T10βOH*, *T13αOH*, *TAT*, *TBT* and *DBAT*) in two plasmids. *In vitro* experiments demonstrated the functionality of the recombinant proteins, and immunoblotting confirmed their *in vivo* expression. GGPP and taxadiene were detected in the system, demonstrating the functionality of the fusion proteins in *S. cerevisiae*, and that the native isoprenoid precursors IPP and DMAPP were sufficient to initiate the terpene biosynthetic pathway (DeJong et al., 2006). Nevertheless, taxa-4(20), 11(12)-dien-5α-ol, the product of the enzyme T5αH, was obtained in very small quantities, comparable to those in *E. coli* (Huang et al., 2001) and much lower than the levels obtained in *Taxus* cell cultures (Ketchum et al., 1999). On the other hand, subsequent taxanes in the PTX pathway could not be detected, indicating restrictions in the metabolic flux at the level of T5αH, which was introduced to increase taxadiene production in the yeast. Also in *S. cerevisiae*, Engels et al. (2008) introduced heterologous genes encoding isoprenoid biosynthetic enzymes from the early steps of taxane metabolism, as well as a regulatory factor to inhibit competitive pathways. The highest taxadiene contents (8.7 mg/L) were obtained when the yeast co-expressed the following genes: a truncated version of 3-hydroxyl-3-methylglutaryl-CoA reductase (*HMG-CoA reductase*) isoenzyme 1, which is not subject to feedback inhibition; a mutant regulatory protein, UPC2-1; the *GGPPS* gene from *Sulfolobus acidocaldarius*, which does not compete with steroid synthesis; and a codon-optimized *TXS* gene to ensure high-level expression. A higher taxadiene production (72.8 mg/L) was achieved by Ding et al. (2014) in a *S. cerevisiae* strain co-expressing the most effective GGPPS gene (selected by protein modeling and a docking strategy from eight different sources), the *erg20* gene (encoding farnesyl diphosphate), a truncated *HMGR* gene, and the *TXS* gene.

Keasling’s group (Nowrouzi et al., 2020) considerably improved taxadiene production in *S. cerevisiae* cell cultures maintained at 22°C by using the promoter GAL1 and an engineered TASY (TXS) variant with a 60-residue truncation. After confirming that chromosomal integration resulted in a higher taxadiene titer than episomal expression, a cassette containing an N-terminal yeast codon-optimized MBP tagged TASY-ERG20* fusion gene was developed with the dual aim of increasing precursor availability and improving TASY solubility. This cassette was chromosomally integrated into two loci to obtain the strain LRS5 in which the taxadiene levels were 57 ± 3 mg/L in a small-scale culture and 129 mg/L in a larger scale culture maintained at 30 °C.

The same research group (Walls et al., 2021) improved the production of taxadiene, taxadiene-5α-ol (T5αol), and taxadien-5-yl acetate (T5αAc) in *S. cerevisiae* LRS6. The strain was constructed in the same way as LRS5 but with the addition of gene sequences encoding *CYP725A4*, its cognate cytochrome P450 reductase (CPR), and the *TAT* gene obtained from *T. cuspidata*. The synthetic genes were codon-optimized for *S. cerevisiae* expression. Besides taxadiene and other products commonly formed when the promiscuous enzyme TXS is active (such as verticillene and isotaxadiene), the strain produced GGOH and other diterpenoids, as well as several oxygenated compounds such as OCT and iso-OCT (5(12)-oxa-3(11)-cyclotaxane and 5(11)-oxa-3(11)-cyclotaxane, respectively), usually found in *E.coli* overexpressing the *CYP725A4* gene (Edgar et al., 2017). The formation of multiple compounds from the same precursors in side reactions partly explains the low production of taxanes in the PTX biosynthetic pathway. In this study, the culture was scaled up and the conditions optimized. Moreover, the pH control was improved, as were the identification and quantification methodologies for the target compounds. When working with a 1 L bioreactor, taxadien‐5α‐yl‐acetate levels were 3.7 mg/L, those of the taxadien‐5α‐ol isomer were about 20 mg/L, and total oxygenated taxane contents increased 2.7‐fold to 78 mg/L, the highest quantity reported in yeast to date.

In a very recent study aimed at increasing the production of the upstream PTX precursors taxadiene, T5αol and T5Ac, Walls et al. (2022) improved the culture conditions of *S. cerevisiae* LRS6 in a 1L BIOSTAT bioreactor. The effects of nutrient stress were identified and resolved by increasing the culture nutrient supply. Taxane accumulation was further improved in a small-scale bioreactor by using a statistical definitive screening design. Finally, in optimum culture conditions in a 1 L bioreactor, the main diterpene product was taxadiene, with a maximum titer of 71 ± 8 mg/L obtained at 95 h of culture, although iso-taxadiene and the side-product verticillene were also found. A further 12 diterpenoid products of the CYP725A4 and TAT enzymes were observed. The major CYP725A4 product was the previously identified potential T5αol isomer diterpenoid 1 with a maximum titer of 97 ± 2 mg/L; iso-OCT, OCT and T5αol were detected in quantities of 16 ± 3, 44 ± 3 and 42 ± 4 mg/L, respectively. The desired TAT product, T5Ac, was obtained with a maximum level of 21 ± 0.3 mg/L, which was almost 6-fold higher than previous maxima. This improvement in taxane levels reflects the significant progress made in optimizing *S. cerevisiae* cultures and indicates their potential for scaling up to industrial scale productivity.

However, the T5αOH gene is generally poorly expressed in heterologous hosts and has low catalytic activity, converting less than 10% of taxadiene to T-5α-ol. Its main products are OCT and its isomer, iso-OCT, which increases the branching of the PTX pathway. As in *E. coli*, achieving the enzymatically active form of the cytochrome P450 in yeast is kinetically limited by reliance on an endogenous NADPH:cytochrome reductase for coupling the electron flow. Further interference is caused by the formation of side-products and the presence of endogenous metabolites. Consequently, production in more amenable, higher biomass-producing and fast-growing heterologous hosts such as plants offers considerable advantages. These include the control and manipulation of metabolic flux by improving enzyme expression, pathway regulation, the availability of cofactors, and engineering competing pathways, as well as the availability of carbon resources from photosynthesis.

### 4.3 Metabolic engineering in model plants

Exploiting the benefits of a model plant with its own source of IPP and plastid DMAPP, parts of the PTX biosynthetic pathway were transferred into *A. thaliana* (Besumbes et al., 2004). After the introduction of the *TXS* gene under the control of the constitutive CaMV 35S promoter, taxadiene was produced in the homozygous plants, indicating that the recombinant protein was functional in *A. thaliana*. However, some deleterious effects were observed, such as reduction in hypocotyl length, leaf discoloration, and reduction in growth and flowering (Besumbes et al., 2004). Possible explanations could be the cellular toxicity of taxadiene, or the constitutively active *TXS* gene interfering with the synthesis of terpenes vital for plant development, such as gibberellins or carotenoids. The negative effects of *TXS* expression were subsequently avoided by placing the *TXS* gene under the control of a glucocorticoid-inducible promoter, thereby preventing metabolic redirection of GGPP into the terpene pathway during plant growth and development (Besumbes et al., 2004). However, although the proportion of deleterious phenotypes was reduced, taxadiene production remained low. Using the same model plant, Khani et al. (2010) expressed the *TXS* gene in *A. thaliana* (ecotype Columbia-0) by transformation with *A. tumefaciens* AGL1 carrying the plasmid pTA-TXS-His. The transformation and transgene expression were confirmed, although the taxadiene production was not measured.

Several studies have also established *Nicotiana* cultures heterologously expressing PTX biosynthetic genes. The use of *Nicotiana* spp. is advantageous due to its high biomass, complex secondary metabolism, and easy cultivation both *in vivo* and *in vitro*. In 2008, Rontein et al. (2008) introduced the *TXS* and *T5αH* genes in wild tobacco (*Nicotiana sylvestris*) after knocking out production of cembratrien-diol. The plant transformation was carried out using *Agrobacterium* LBA4404 carrying taxa-4(5),11(12)-diene synthase and *CYP725A4* genes for specific expression in trichome cells. However, instead of T-5α-ol, the transformed plants produced a cyclic ether, 5(12)-oxa-3(11)-cyclotaxane (OCT), as occurs in *E. coli* and *S. cerevisiae* overexpressing the same genes.

*Nicotiana benthamiana* plants ectopically expressing the TXS gene produced up to 27 μg/g dry weight of taxadiene (Hasan et al., 2014). Leaf discs of *N. benthamiana* were transformed with *A. tumefaciens* and the taxadiene contents in the resulting homozygous plants were increased by elicitation with MeJa. However, the highest level of taxadiene (up to 50 µg/g dry weight) was obtained after silencing the phytoene synthase (PSY) and phytoene desaturase (PDS) genes, which divert GGPP towards tetraterpene biosynthesis instead of leaving it free for the synthesis of diterpenes such as paclitaxel.

More recently, (Li et al., 2019) transiently transformed *N. benthamiana* by co-infiltration with different strains of *A. tumefaciens* GV3101. A partial PTX biosynthetic pathway was engineered in *N. benthamiana* by compartmentalizing TXS and the T5H-CPR fusion construct (*T5H* and *CPR* encoding taxadiene 5α-hydroxylase, and cytochrome P450 reductase, respectively) in chloroplasts, using the native TXS signal peptide as a chloroplast-targeting peptide. Combining this compartmentalization strategy with enhanced precursor availability and channeling carbon towards taxane biosynthesis by co-expression of *DXS* (1-deoxy-D-xylulose-5-phosphate synthase) and *GGPPS* (GGPP synthase) genes resulted in the accumulation of relatively high levels of taxadiene (56.6 ± 3.2 μg g^−1^ fresh weight), and most importantly, taxadiene-5α-ol (1.3 ± 0.5 μg g^−1^ fresh weight). It is clear that the inter-organellar transport of taxane intermediates is a major barrier in plant PTX production systems, blocking the access of CYP450s in the endoplasmic reticulum to plastid-localized diterpenoid substrates.

As TXS is a chloroplast enzyme, Fu et al. (2021) established transplastomic *N. tabacum* cv. Petit Havana with the aim of producing taxadiene in these organelles. Surprisingly, the levels obtained in plants expressing the *TXS* gene in the chloroplast were very low, as were those in the cytosol. However, when the TXS carrying a chloroplast transit peptide was expressed in the nucleus, taxadiene in the chloroplast increased significantly. The results demonstrated that the transport of this intermediate to the chloroplast and its post-translational modifications is important for high levels of taxadiene formation.

Despite the important advancements described here, T5αH remains one of the main bottlenecks in the PTX biosynthetic pathway, due to its poor activity in generating T5αol and its facility to produce side-products that compete for the same precursor. Several synthetic biology tools have been leveraged to optimize T5αH expression and activity, including truncations, promoter optimization, CPR optimization, compartmentalized engineering in plant organelles, and the use of riboregulated switchable feedback promoters (rSFPs). Computational and experimental approaches have also been used to provide new insights into the catalytic mechanisms of TXS and T5αH (Mutanda et al., 2021).

Engineering PTX production in heterologous hosts is a promising but also highly challenging task. Besides all the difficulties outlined here, many of the biosynthetic enzymes involved have not been elucidated, which prevents the heterologous reconstitution of the entire pathway (Courdavault et al., 2020). Other gaps in our understanding of PTX metabolism include the regulatory and control mechanisms, which also limits synthetic biology approaches to its production.

Very recently, Xiong et al. (2021) completed the chromosome-level genome of *T. chinensis* var. mairei. Their study revealed that several PTX biosynthetic genes share the same chromosomal location and identified a gene cluster expressing *TXS* and *T5αH*, activated by jasmonates. They also discovered different genes encoding enzymes with the same function, but with different regulation. The new knowledge generated by this research and previous metabolic studies, together with the application of computational tools, will facilitate the discovery of missing steps in PTX biosynthesis and its regulation. In this way, the potential of biotechnological systems for large-scale production of this vital anti-cancer drug may be realized.

## 5 Challenges and perspectives for taxane production

The obstacle that has remained during decades of studies with the *Taxus* spp. is undoubtedly the difficulty of its genetic transformation, followed by its poor adaptation to *in vitro* systems. These obstacles have led to an intense search for heterologous systems that are more friendly to transformation and cultivation techniques. But these systems at the same time have revealed the hidden complexity behind the synthesis of taxanes. Speaking of heterologous plant systems, the wide variety of competitive pathways or enzymes capable of metabolizing taxane precursors represents the greatest challenge. For the remaining eukaryotic systems, the lack of knowledge of all the biosynthesis steps as well as solubility problems of the heterologous proteins used to recreate the synthesis of taxanes represent the biggest task. In terms of prokaryotic cells, the absence of numerous metabolic pathways necessary to generate the precursors, the low solubility of heterologous proteins, and the potential toxicity of the generated compounds are the main obstacles. With the recent sequencing of the *Taxus* spp. genome as well as advances in enzymatic engineering using computational tools (Mutanda et al., 2021), the biotechnological production of taxanes will mainly favor heterologous systems.

## Author contributions

EP-M, MB, and JP: Conceived of the presented idea; EP-M and DH-M: Writing—original draft preparation; MAA and AE: Figure processing and data gathering; MB, JP, DH-M, and EM: review and editing. All authors contributed to the article and approved the submitted version.
